# Setting the standards for safeguarding health and wellbeing in US Immigration and Customs Enforcement detention facilities

**DOI:** 10.1016/j.lana.2024.100851

**Published:** 2024-08-01

**Authors:** Rohan Borschmann, Stuart A. Kinner, Kyli Hedrick

**Affiliations:** a Centre for Mental Health and Community Wellbeing, Melbourne School of Population and Global Health, University of Melbourne, Melbourne, Australia; b Medical Sciences Division, Department of Psychiatry, University of Oxford, Oxford, UK; c Oxford Health NHS Foundation Trust, Oxfordshire, UK; d Justice Health Group, enAble Institute, Curtin University, Perth, Australia; e Centre for Adolescent Health, Murdoch Children's Research Institute, Royal Children's Hospital, Melbourne, Australia; f Griffith Criminology Institute, Griffith University, Brisbane, Australia; g Melbourne School of Population and Global Health, University of Melbourne, Melbourne, Australia

The United States (US) Immigration and Customs Enforcement (ICE) detention network is the largest national immigration detention system in the world, detaining almost 38,000 people on any given day.^1^ ICE “detain [s] non-citizens to secure their presence for immigration proceedings, or their removal from the US”^1^; this includes asylum seekers found to have credible claims for protection, women and children escaping violence, and long-term US residents—with or without legal status—with strong existing ties to the US, among other vulnerable individuals.^2^ Although ICE detention is purported to be non-punitive and for administrative purposes only,^1^ and most people detained in ICE custody have no criminal record,^3^ 90.8% are held in private, for-profit detention facilities run by prison operators contracted by ICE.^1^ Despite this, and contrary to repeated calls to implement community-based alternatives to immigration detention,^4^ the recently introduced 2024 Homeland Security appropriations bill^5^ will further increase ICE funding in order to manage a projected daily immigration detainee population of 41,500 in the financial year 2024–25, at a cost of $3.55 billion USD.^6^

A large and growing evidence base has highlighted the adverse conditions, detrimental policies, and harmful practices associated with immigration detention in multiple countries,^3^^,^^7^ including in relation to US ICE settings.^8^ There is strong evidence that immigration detention has an adverse impact on the mental health of those detained,^7^^,^^9^ and that conditions in ICE detention facilities have contributed to numerous preventable deaths in recent years.^10^ Research has highlighted the vulnerabilities and poor health profiles of many people held in immigration detention^11^ and there have been documented reports of abhorrent human rights abuses occurring in ICE detention.^2^ Within this context, Annette Dekker and colleagues’ Viewpoint^12^ critiques the existing standards for healthcare provision in ICE detention settings and documents evidence of grossly inadequate mental healthcare, inappropriate and excessive use of solitary confinement, and an 11-fold increase in the suicide rate in ICE detention between 2010 and 2020. Dekker and colleagues provide a much-needed call to action to address the lack of (1) reporting and transparency of ICE healthcare standards, and (2) accountability of healthcare providers in these facilities.

In their examination of healthcare standards in ICE detention settings, Dekker and colleagues scrutinise the 2016 revised Performance-Based National Detention Standards (PBNDS) that apply to facilities exclusively housing detained immigrants, and which are largely run by private, for-profit prison operators.^1^^,^^12^ These federally funded facilities currently detain 80% of the average daily ICE detention population.^1^ The authors highlight the unacceptably vague standards in the PBNDS, and the striking absence of specific guidelines to which facilities must adhere. They also highlight serious flaws in PBNDS compliance inspections, including a lack of transparent information about how deficiencies are identified, addressed, and assessed. This opacity is compounded by remarkable variation in how the PBNDS are applied between detention facilities.^2^ Dekker and colleagues also note the absence of formal standards regarding which health metrics should be monitored, and a lack of quality monitoring and accountability of publicly-funded healthcare outcomes in these facilities. This contributes to a system in which deficiencies in the provision of healthcare are frequently overlooked and, even when identified, result in neither improved conditions nor accountability.

Dekker and colleagues’ concerns mirror those documented in relation to Australian immigration detention for more than 30 years.^13^ Although all individuals in places of detention—including immigration detention—retain the right to the highest attainable standard of physical and mental health,^14^ in practice these rights have been diminished in both ICE and Australian detention facilities.^13^ In the absence of abolishing immigration detention, Dekker and colleagues argue that reporting of health metrics in detention must be frequent, timely, granular, monitored by an independent body, and publicly reported. We agree and, in addition to these US-based suggestions, we recommend (1) developing tools to routinely assess progress against standardised health outcome measures, and (2) the establishment of an independent panel of clinical experts (including psychologists, psychiatrists, public health experts, dentists, nurses, and other clinicians) to monitor and report regularly on healthcare provision in immigration detention.^13^ Ideally, such a panel would be authorised to provide ongoing monitoring and reporting on culturally appropriate, trauma-informed healthcare provision. A mechanism to ensure that particularly at-risk and/or vulnerable individuals are released on medical or mental health grounds should also be developed and implemented by this independent panel. Meaningful consequences must be enforced for contractors who violate standards, including for facilities following any deaths.^10^

Dekker and colleagues' Viewpoint draws attention to the ongoing, serious concerns regarding the health and human rights of individuals in immigration detention,^2^, ^3^, ^4^^,^^7^^,^^8^ and highlights the ways in which federally funded—but privately operated—prison operators may profit^2^^,^^3^ while evading public scrutiny for any harms perpetrated.^2^ With the governments of many countries around the world seeking to outsource and externalise their legal and human rights responsibilities to asylum seekers,^15^ the authors' investigation into the US immigration detention system should serve as a cautionary tale. Our own recent research,^15^ which examined the Australian government's costly use of controversial private prison contractor Management and Training Corporation (MTC)—against a backdrop of multiple serious and ongoing allegations in the US—for the new ‘enduring form of offshore processing’ in the Pacific Island nation of Nauru, highlighted legitimate concerns for the health and wellbeing of current and future asylum seekers detained in MTC-run facilities. Our concerns regarding the risk of preventable psychological and physical harm were further heightened by the lack of transparency and accountability associated with such contractual arrangements, including in relation to the provision of healthcare, and the fact that the Australian government has implemented permanent immigration detention arrangements.^15^

In the absence of abolishing immigration detention, the timely provision of evidence-based, trauma-informed, and culturally appropriate healthcare services (with interpreter access where necessary) in all ICE detention centres is critical for preventing further harm and ensuring that all people detained in such facilities retain the right to the highest attainable standard of physical and mental health. Unfortunately, as Dekker and colleagues demonstrate so unambiguously, ICE—and all those it contracts—currently falls drastically short of meeting these standards.

## Contributors

KH and RB wrote the first draft of this Comment. SK provided critical input to subsequent versions, along with KH and RB. All authors approved the final version.

## Declaration of interests

KH is the Director of Community-Minded Psychological Services, a private practice for people from immigrant, asylum seeking, and refugee backgrounds, which has received funding from the Australian government to provide psychological services to asylum seekers. KH receives personal fees from Settlement Services International to provide clinical assessment and psychological support for asylum seekers released from Australian immigration detention. KH has previously received personal fees from the Victorian Foundation for Survivors of Torture to provide psychological support to people from asylum seeking and refugee backgrounds, and from the Australian government's Department of Home Affairs to conduct independent mental health assessments and provide psychological reports for asylum seekers medically evacuated from offshore immigration detention. She has also received personal fees from the Nordic Refugee Determination: Advancing Data Science in Migration Law project, funded by Nordforsk. The views expressed here are her own, and those of her co-authors. RB and SK declare no competing interests.
